# Synthesis and Characterization of N-Doped Porous TiO_2_ Hollow Spheres and Their Photocatalytic and Optical Properties

**DOI:** 10.3390/ma9100849

**Published:** 2016-10-19

**Authors:** Hongliang Li, Hui Liu, Aiping Fu, Guanglei Wu, Man Xu, Guangsheng Pang, Peizhi Guo, Jingquan Liu, Xiu Song Zhao

**Affiliations:** 1 Institute of Materials for Energy and Environment, Qingdao University, Qingdao 266071, China; boluo.666@163.com (H.L.); wuguanglei@qdu.edu.cn (G.W.); pzguo@qdu.edu.cn (P.G.); chezxs@qdu.edu.cn (X.S.Z.); 2 Laboratory of New Fiber Materials and Modern Textile, Growing Basis for State Key Laboratory, College of Materials Science and Engineering, Qingdao University, Qingdao 266071, China; jliu@qdu.edu.cn; 3 State Key Laboratory of Inorganic Synthesis and Preparative Chemistry, College of Chemistry, Jilin University, Changchun 130012, China; xuman.jlu@aliyun.com (M.X.); panggs@jlu.edu.cn (G.P.); 4 School of Chemical Engineering, The University of Queensland, St Lucia, Brisbane QLD 4072, Australia

**Keywords:** mesoporous TiO_2_ hollow spheres, MF microsphere template, photocatalytic properties, optical properties

## Abstract

Three kinds of N-doped mesoporous TiO_2_ hollow spheres with different N-doping contents, surface area, and pore size distributions were prepared based on a sol–gel synthesis and combined with a calcination process. Melamine formaldehyde (MF) microspheres have been used as sacrificial template and cetyltrimethyl ammonium bromide (CTAB) or polyvinylpyrrolidone (PVP) was selected as pore-directing agent. Core–shell intermediate spheres of titania-coated MF with diameters of 1.2–1.6 μm were fabricated by varying the volume concentration of TiO_2_ precursor from 1 to 3 vol %. By calcining the core–shell composite spheres at 500 °C for 3 h in air, an in situ N-doping process occurred upon the decomposition of the MF template and CTAB or PVP pore-directing surfactant. N-doped mesoporous TiO_2_ hollow spheres with sizes in the range of 0.4–1.2 μm and shell thickness from 40 to 110 nm were obtained. The composition and N-doping content, thermal stability, morphology, surface area and pore size distribution, wall thickness, photocatalytic activities, and optical properties of the mesoporous TiO_2_ hollow spheres derived from different conditions were investigated and compared based on Fourier-transformation infrared (FTIR), SEM, TEM, thermogravimetric analysis (TGA), nitrogen adsorption–desorption, and UV–vis spectrophotoscopy techniques. The influences of particle size, N-doping, porous, and hollow characteristics of the TiO_2_ hollow spheres on their photocatalytic activities and optical properties have been studied and discussed based on the composition analysis, structure characterization, and optical property investigation of these hollow spherical TiO_2_ matrices.

## 1. Introduction

With the increasing severity of global environment pollution and energy shortage, titania-based photocatalytic materials with unique characteristics in structure and band position are currently one of the fastest-growing areas of materials research. It has been widely investigated as an important material with novel physical properties, chemical stability, environmental compatibility, and low production cost [1,2,3,4]. Nevertheless, the properties and potential applications are dependent on structure, morphology, crystalline size, and composition of the TiO_2_ particles. Consequently, considerable explorations have been focused on two concepts, namely structure engineering and bandgap engineering [5]. Due to the novelty in structures, and thus the derived properties, fabrications of hollow TiO_2_ materials have attracted much attention during the last few decades and various methods for the preparation of hollow spherical structures have been developed [6,7]. In particular, the template-sacrificial technique is one of the most widely used and effective methods for the fabrication of hollow structures [8]. To date, the structure-directing templates, including polystyrene latex particles, resin spheres, carbon nanoparticles, vesicles, and gas bubbles have been extensively investigated [9,10,11].

Mesoporous-structured TiO_2_ with a large surface area and porous frameworks could show more effective photocatalytic activity as compared with the bulk ones [12]. The combination of porous character with a hollow property may endow the TiO_2_ materials with additional novel optical properties. It has also been reported that the doping of the TiO_2_ crystallites with heteroatoms could narrow its bandgap and shift its optical response from the ultraviolet to the visible-light region. Therefore, a TiO_2_ hollow structure with mesoporous shell and doped with heteroatoms is desirable for the photocatalytic performance [13,14,15]. Nitrogen-doped TiO_2_ materials have been obtained using ammonia or N-containing additives as N sources [16,17,18], yet little is known about the nitrogen-doped TiO_2_ hollow spheres prepared by using hard spheres as both the core templates and the nitrogen source. On the other hand, in most of studies the enhancement of photocatalytic activities of the N-doped TiO_2_ have been attributed to the introduction of N heteroatoms, however, the influencing mechanism of N-doping on the photocatalytic property is still a controversial issue [19]. Some reports demonstrated that the doping of N atoms could introduce interbands or defects, which then can enhance the light absorption ability of the TiO_2_ particles or increase the electron-hole separation efficiency, resulting in the increase of their photocatalytic activities [20,21]. Other reports proposed that the doping of heteroatoms could narrow the bandgap of the doped TiO_2_ materials in comparison with the pristine one [22]. In principle, the surface area and channel inside the catalytic particles also play a very important role in determining the kinetic characteristic of the catalyst. Thus, in some cases specific surface area and porosity of the TiO_2_ materials can significantly influence their photocatalytic properties, whereas the doping process sometimes will affect the particle size and the porous properties of the TiO_2_ catalysts. It has also been revealed that the exposed lattice facets of the TiO_2_ crystals could influence obviously on their photocatalytic performance [23,24].

In this study, we report on the preparation of N-doped TiO_2_ hollow spheres with porous walls of variable thickness using melamine formaldehyde (MF) microspheres as sacrificial template through a sol–gel synthesis and a calcination process. Cetyltrimethyl ammonium bromide (CTAB) surfactant or polyvinylpyrrolidone (PVP) polymer has been exploited as a pore-directing agent based on the electrostatic assembly technique. Both the MF template and pore-directing agent also plays a role as N-sources, and therefore in situ N-doping of TiO_2_ is achieved during the calcinations process. For comparison, hollow TiO_2_ spheres as control sample were also fabricated with a similar procedure but without the surfactant. The influence of N-doping on their visible-light-driven photocatalytic activities and their optical properties of these three kinds of porous TiO_2_ hollow spheres have been investigated and compared in detail. The effect of the porous character on their optical properties of mesoporous TiO_2_ hollow spheres has also been studied through a UV–visible spectrometer and analyzed based on the theory. 

## 2. Experimental Section

### 2.1. Materials

Formaldehyde (37%), melamine, polyvinyl alcohol (PVA), acetic acid, absolute ethanol, cetyltrimethyl ammonium bromide (CTAB), polyvinylpyrrolidone (PVP), ammonia (25%–28%), tetrabutyl titanate (Ti(OC_4_H_9_)_4_, TBOT), Degussa P25, and methylene blue (MB) were used. All the chemicals were of analytical reagent (AR) grade and used without further purification. Distilled water was used in the experiments.

### 2.2. Synthesis of Monodisperse MF Microspheres

Firstly, a solution of formaldehyde (5.3 mL, 37%) was mixed with melamine (2.8 g) under stirring at 60 °C for 20 min to obtain a clear precursor solution. When the resulting precursor solution was cooled down to 35 °C, then poured it into 90 mL of PVA aqueous solution (0.4 wt %), obtaining a mixture. Consequently, acetic acid was introduced into the mixture until the pH value was adjusted to 4.2 and the solution was then kept stirring at 65 °C for 30 min, obtaining colloidal MF particles. White MF spheres were collected by centrifugation, washed three times with water and ethanol, respectively, and finally dried in air at 60 °C.

### 2.3. Coating the MF Microspheres with Titania

As-prepared MF microspheres (0.2 g) was dispersed homogenously into a 100 mL of absolute ethanol by sonicating the mixture for 15 min with a high-power sonicator. Then the MF ethanol suspension was transferred into a round-bottomed flask, and a solution containing 1 mL of NH_4_OH, 1 mL of water, and 0.06 g of CTAB was added to the MF suspension solution at room temperature under magnetic stirring. Thereafter, a 1.0 vol % TBOT ethanol solution (0.15 mL TBOT dissolved into 15 mL ethanol) was added to the above mixture dropwise, and the reaction was allowed to sustain for 6 h at 40 °C under continuous stirring. Solid was then separated from the suspension by centrifugation and washed three times with distilled water and ethanol, respectively. The solid was redispersed into an appropriate volume of deionized water and cooled to freeze. White powder denoted as MF@C-TiO_2_ was then obtained after a 24 h freeze-drying process on a FD-1A-50 freeze-dryer and was preserved for the subsequent experiments. 

For comparison, PVP was used instead of CTAB as pore-directing agent to prepare the core-shell structured composite microspheres with the same procedure as described above, and white powders denoted as MF@P-TiO_2_ was obtained and preserved for the subsequent experiments. Core–shell structure of MF@B-TiO_2_, which contains no pore-directing agent, was also prepared as control sample using the same procedure in the absence of surfactant. 

### 2.4. Preparation of TiO_2_ Hollow Spheres

TiO_2_ hollow microspheres, designated as C-TiO_2_, P-TiO_2_, and B-TiO_2_ were obtained by calcining the MF@C-TiO_2_, MF@P-TiO_2_, and MF@B-TiO_2_ composites to remove the MF cores and the pore-directing agents. The thermal treatments were performed with a programmed muffle furnace at 500 °C for 3 h in air with a heating rate of 1 °C/min.

### 2.5. Photocatalytic Reaction

The photocatalytic activity of the X-TiO_2_ hollow spheres (B-TiO_2_, P-TiO_2_, C-TiO_2_) and the commercial Degussa P25 particles, which are considered as one of the most efficient commercial titania with relatively high catalytic properties, were investigated with 30 mg of samples, respectively, suspended in a 200 mL of MB aqueous solution (0.0375 mmol/L). The suspensions were sonicated in the dark for about 30 min to mix the catalyst homogeneously with MB, and then they were irradiated by a 300 W medium-pressure mercury lamp with mimic visible light (wavelength >420 nm). The amount of residual MB in the solution at different time intervals were monitored by measuring the absorption of MB at wavelength of 665 nm using a UV–vis spectrophotometer, and then converted the absorption intensity into the mass of methylene blue. 

### 2.6. Characterization

Fourier-transformation infrared (FTIR) were carried out with a Nicolet 5700 FT-IR spectrometer (Thermo Scientific, Madison, WI, USA) on KBr pellets. The thermogravimetric analysis was performed with a Mettler Toledo TGA-2 thermal gravimetric analyzer (Mettler-Toledo GmbH, Greifensee, Switzerland). The morphologies of the samples were examined by a JEOL JSM-7800F scanning electron microscope (SEM) (JEOL, Akishima, Japan) and a JEM-2100 transmission electron microscope (TEM) (JEOL, Akishima, Japan). Powder X-ray diffraction (XRD) patterns of the samples were collected using a Rigaku Ultima IV X-ray diffractometer (Rigaku Corporation, Akishima-Shi, Japan) equipped with graphite monochromatized Cu Kα radiation (λ = 0.15418 nm). The specific surface areas were estimated with the Brunauer–Emmett–Teller (BET) method with N_2_ adsorption data in the relative pressure range of *P*/*P*_0_ = 0.05–0.35 and the pore size distributions were calculated using the Barrett–Joyner–Halenda (BJH) model applied to the desorption branch of the N_2_ isotherms obtained with a TriStar 3000 Surface Area and Pore Analyzer (Micromeritics, Norcross, GA, USA). The XPS data were accumulated on an AXIS HS (Kratos Analytical, Manchester, UK) electron spectrometer system with a monochromatized Al Kα standard X-ray source. The binding energies were calibrated by referencing the C1s to 285.0 eV. UV–vis spectra were measured through a TU-1901 UV-Visible spectrophotometer (Purkinje General Instrument Co., Ltd., Beijing China). 

## 3. Results and Discussion

### 3.1. FTIR Analysis

**Figure 1:**
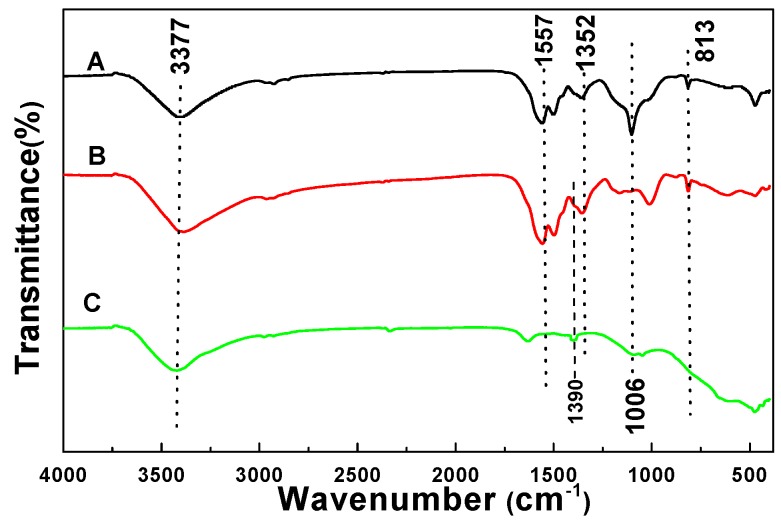
Fourier-transformation infrared (FTIR) spectra of **A** pure melamine formaldehyde (MF) microspheres, **B** MF@C-TiO_2_ core-shell microspheres and **C** the resulting C-TiO_2_ hollow microspheres.

Figure 1 shows the FTIR spectra of the pristine MF spheres, MF@C-TiO_2_ core–shell composites, and the corresponding C-TiO_2_ hollow spheres. Both curve A and curve B showed the characteristic absorption bands of MF at about 3377, 1557 (1492, 1352), 1166, 1006, and 813 cm^−1^, which originate from the vibrations of hydroxyl/amino (–OH/–NH_2_), amino (–NH_2_), amine (C–N), ether (C–O–C), and C–N–C groups, respectively. All the MF characteristic bands disappear in curve C, which indicates the successful removal of the MF cores during calcinations. A weak peak, which may be the signal of incorporated nitrogen in C-TiO_2_ sample, appears at 1390 cm^−1^ in curve C [25]. The weak peak located at around 1630 cm^−1^ is typical of CO_2_ absorption in air. The FTIR results support the formation of the MF@C-TiO_2_ core–shell composite intermediate after the sol–gel deposition process and the formation of the hollow TiO_2_ product after calcinations. 

### 3.2. Thermogravimetric Analysis 

**Figure 2:**
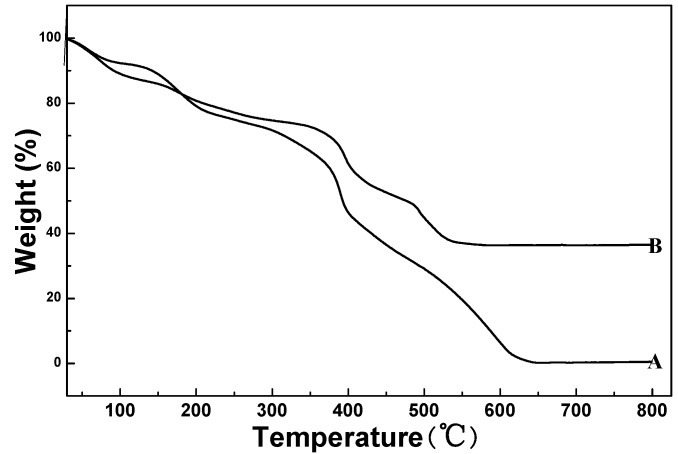
Thermogravimetric analysis (TGA) curves of pure MF sphere template **A**, and the as-dried core–shell structured spheres of MF@C-TiO_2_ **B**.

The results of thermal analysis for the pure MF spheres and the MF@C-TiO_2_ core–shell composite spheres are illustrated in Figure 2. From the figure, one can see that the pure MF spheres and the MF@C-TiO_2_ composites showed quite similar weight loss behavior and there are three main stages of weight loss in both the curves. The first weight loss in the range of 50–100 °C may be the desorption of absorbed water on the surfaces of the samples, whereas the second weight loss between 100 and 250 °C is attributed to the dehydration and densification of the MF templates and titanium precursors. The ultimately rapid weight losses from about 350 °C can be assigned in curve A to the burning of the MF spheres and that in curve B to the combination of the burning of MF templates and the CTAB pore-directing agents, and also the condensation of the titania in the composite spheres. In comparison of curve A with curve B it can be derived that the weight percentage of titania in the MF@C-TiO_2_ composite is about 40%. The TGA results also provide the information for selecting calcinations temperature to remove the MF core from the core–shell composites, and temperature increasing rate to avoid the breaking of the hollow products.

### 3.3. SEM and TEM Analysis

**Figure 3:**
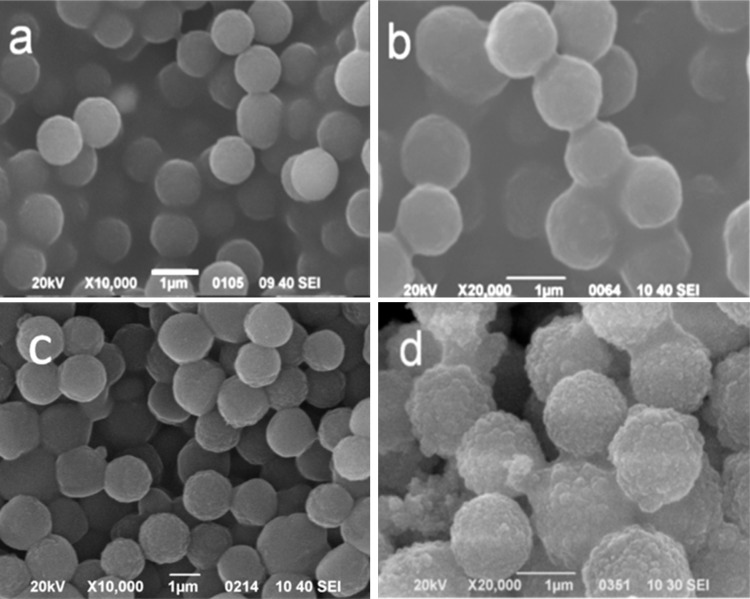
SEM images of (**a**) MF template microspheres and the MF@C-TiO_2_ intermediate spheres derived from tetrabutyl titanate (TBOT) ethanol solution of different concentrations: (**b**) 1 vol %; (**c**) 2 vol %; (**d**) 3 vol %.

Figure 3a illustrated the SEM image of the MF template microspheres, and it can be seen that the MF templates showed a smooth surface texture with relative uniform size of about 1 μm. Figure 3b–d showed the SEM images of the MF@C-TiO_2_ composite microspheres fabricated with different contents of TBOT. From the figure, one can clearly see that the mean diameter of the core–shell composite increases obviously with the increasing of the TBOT content in the ethanol solution from 1 to 3 vol %. Meanwhile, the smooth surface of the composite microspheres turns to a rough one. It can also be seen that separated agglomerates of titania spheres were formed with the increasing of the TBOT concentration (Figure 3d). The increase of the average diameter and the surface roughness of the composite microspheres with the increase of TBOT content disclosed that a titania shell had been coated onto the core of MF microspheres during the in situ hydrolysis, condensation, and coating process. A formation mechanism of the titania coating can be described as follows: the surfactant CTAB in the water–ethanol–ammonia mixture hydrolyzed to afford cationic CTA^+^, which then self-assembled to form micelles. The negatively charged ≡TiO^−^ species in ammonia conditions were adsorbed by the CTA^+^ species [26], which formed CTAB/TiO_2_ grains with negatively charged surface, and the grains self-assembled again onto the surface of the positively charged MF spheres, forming TiO_2_-packed MF core–shell composite spheres (MF@C-TiO_2_) via an electrostatic adsorption.

**Figure 4:**
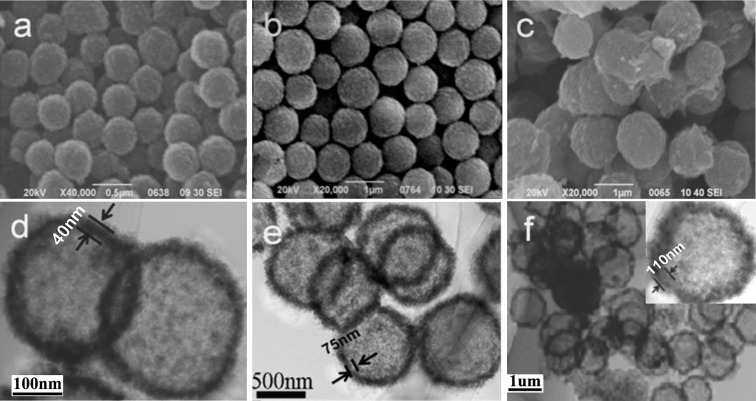
SEM (**a**–**c**) and TEM (**d**–**f**) images of TiO_2_ Hollow spheres derived from MF@C-TiO_2_ composites made with TBOT ethanol solution of different concentrations, (**a**,**d**): 1 vol %; (**b**,**e**): 2 vol %; and (**c**,**f**): 3 vol %, after calcinations.

The size and morphology of the C-TiO_2_ hollow spheres derived from the corresponding MF@C-TiO_2_ composites by calcinations were studied with SEM and TEM, and the images are depicted in Figure 4. It is very interesting that C-TiO_2_ hollow spheres with mean diameters of about 400 nm (Figure 4a,d), 800 nm (Figure 4b,e), and 1.2 μm (Figure 4c,f), were obtained, respectively, after calcinations. Even though the same sized MF microspheres were utilized as templates and mixed with the same volume of TBOT ethanol solution with different TBOT contents (1 vol %, 2 vol %, and 3 vol %, respectively). The shrinkage of the hollow C-TiO_2_ spheres after the calcinations treatments, especially the sample made from low concentration of TBOT can be explained as being due to the dehydration of the MF templates and the densification of the loose outer coating shell, which caused the formation of a condensed titania wall finally. The formation of the hollow spheres with a porous wall after calcinations has been confirmed by TEM measurements. From the TEM images one can also see that the thickness of the shells of the hollow spheres varied with the increasing of the TBOT contents and turned from 40 to 110 nm. The different shrinkage ratios of the MF@C-TiO_2_ core–shell spheres derived from different contents of TBOT indicated that the core–shell spheres with a thicker shell maintained higher thermal stability during the calcination process.

**Figure 5:**
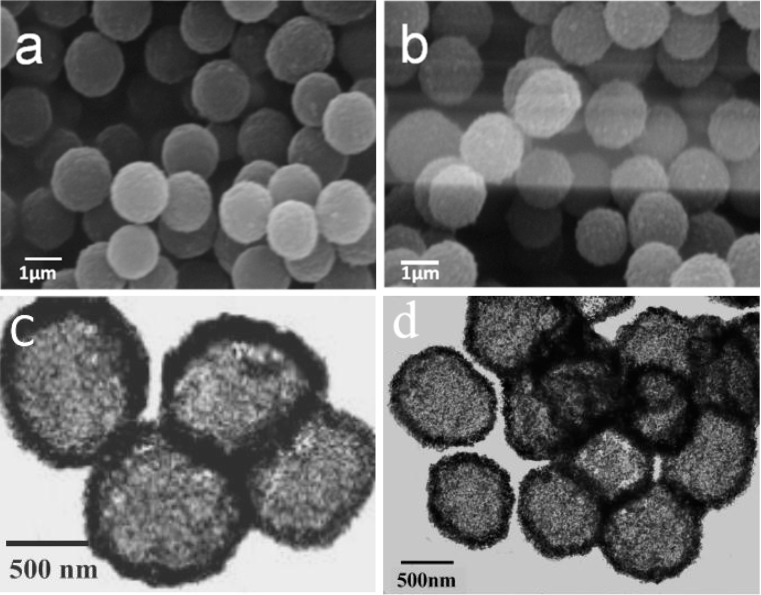
SEM images of MF@B-TiO_2_ (**a**) and MF@P-TiO_2_ (**b**) core–shell structures obtained with 3 vol % of TBOT ethanol solution and the TEM images of B-TiO_2_ (**c**) and P-TiO_2_ (**d**) hollow spheres derived from the corresponding core–shell structures by calcinations.

The SEM images of the MF@B-TiO_2_ and the MF@P-TiO_2_ core–shell spheres are depicted in Figure 5. One can see that the SEM images of the two kinds of core–shell composite spheres are similar in shape to the MF@C-TiO_2_ spheres. The typical TEM micrographs of B-TiO_2_ and P-TiO_2_ derived, respectively, from MF@B-TiO_2_ and MF@P-TiO_2_ core–shell composites intermediate by calcination are illustrated in Figure 5c,d. The strong contrast between the edge and center obviously confirms the hollow nature of the titania spheres, indicating the resulting microspheres were composed of a hollow inner cavity and a thin outer shell as the C-TiO_2_ hollow microspheres. From the microscopy measurements, one cannot see essential differences between the three hollow spheres of C-TiO_2_, P-TiO_2_, and B-TiO_2_, even though their sizes are a bit different.

**Figure 6:**
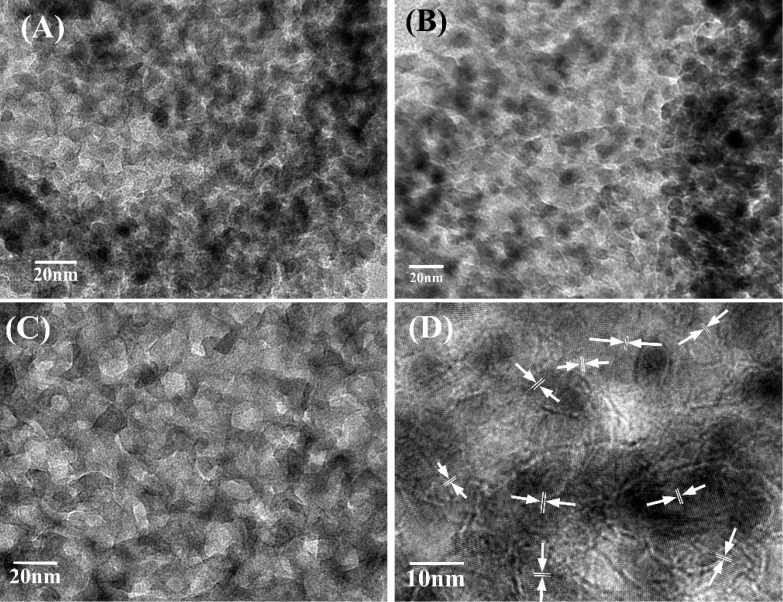
High-resolution TEM (HRTEM) overview images of B-TiO_2_ (**A**); P-TiO_2_ (**B**); and C-TiO_2_ (**C**); and an enlarged image of C-TiO_2_ (**D**) with the arrows in the picture showing the lattice fringes.

Shown in Figure 6A–C are the high-resolution TEM images of C-TiO_2_, P-TiO_2_, and B-TiO_2_ samples, respectively. The images show that all the hollow spheres contain nanoparticles with size of about 5–12 nm and there are no obvious differences between the individual nanoparticles composed of the three different hollow spheres. From the images, one can clearly see the pores in the shells of the three kinds of hollow spheres. Obviously, the pores in the C-TiO_2_ are more regular than those in B-TiO_2_ and P-TiO_2_ spheres. In the high-magnification image of C-TiO_2_ (Figure 6D), the lattice fringes are clearly observable (see the arrows in Figure 6D), confirming their crystallinity, and the same lattice space with a value of about 3.5 Å was calculated for several randomly selected nanocrystals in the shell, which corresponds to the (101) plane of the body centered tetragonal phase of TiO_2_ [27]. As is shown in Figure 6D, some stacking faults are present for the TiO_2_ nanocrystals, which have also been observed previously in TiO_2_ nanoparticles prepared by other methods [28].

### 3.4. XRD Analysis

**Figure 7:**
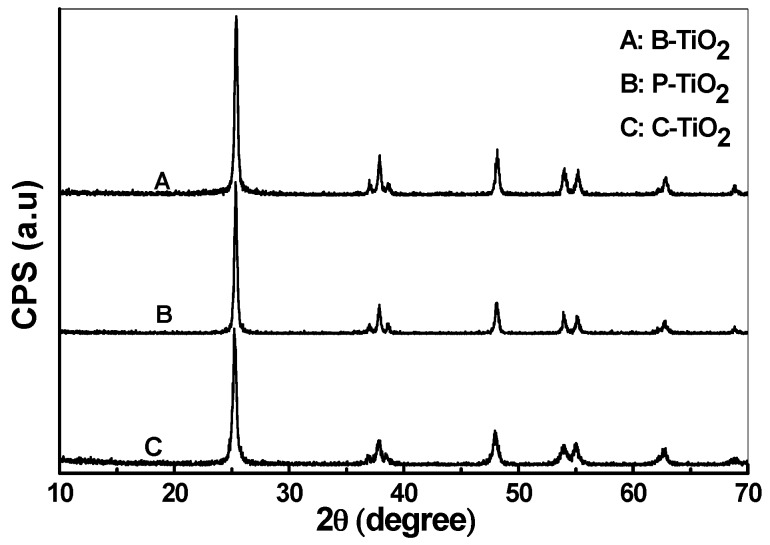
X-ray diffraction (XRD) patterns of the three TiO_2_ hollow spheres.

The XRD patterns of B-TiO_2_, P-TiO_2_, and C-TiO_2_ hollow spheres are presented in Figure 7. The major diffraction peaks observed at 2θ = 25°, 38°, 48°, 54°, and 55° can be assigned as (101), (004), (200), (105), and (211) reflection lines of the body-centered tetragonal phase of TiO_2_ (PDF No. 73-1764). It indicates that all diffraction peaks, peak intensities, and cell parameters of TiO_2_ nanocrystals match well with the standard pattern of anatase phase. The peaks are broadened, showing that the size of the particles is small, as confirmed by the TEM measurements.

To obtain more quantitative information of the TiO_2_ nanocrystals that comprise the hollow spheres, Debye–Scherrer formula, *L* = 0.89λ/*B*cosθ, has been applied to estimate the size of the nanocrystals, where *L* is the average size of the TiO_2_ nanocrystals which constitutes the hollow spheres, *B* is the full-width at half-maximum (FWHM) of the peak in radian, λ is the wavelength of the X-ray radiation, and θ is the angle of diffraction. The values of L obtained for samples B-TiO_2_, P-TiO_2_, and C-TiO_2_ are 21, 24, and 18 nm, respectively, which are a little bit larger than the values derived from the TEM images.

### 3.5. Nitrogen Adsorption–Desorption Analysis

**Figure 8:**
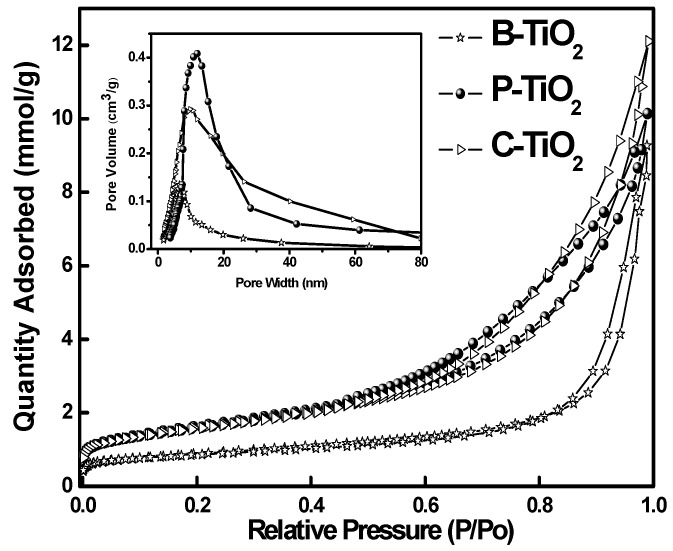
Nitrogen adsorption–desorption isotherms and the corresponding pore-size distributions (the inset) of X-TiO_2_ (X = B, P, C) samples made with equivalent amount of TBOT (3 vol %).

Figure 8 shows the nitrogen adsorption and desorption isotherms of the three kinds of hollow titania spheres. All the three samples show a wide hysteresis loop in the relative high-pressure range of 0.6–1.0, they are the classic type-IV physisorption isotherms of mesoporous structure [29], indicating the presence of pores in the wall of the hollow spheres. The BET specific surface area and pore volume of B-TiO_2_, P-TiO_2_, and C-TiO_2_ hollow titania spheres are calculated to be 98, 128, 131 m^2^/g and 0.13, 0.30, 0.41 cm^3^/g, respectively. These values are larger than that of the P25 TiO_2_ with a BET surface area of about 50 m^2^/g. The inset in Figure 8 shows the pore size distributions of mesoporous B-TiO_2_, P-TiO_2_, and C-TiO_2_ hollow spheres, respectively, and pore sizes centered at about 8.7, 10.6, and 12.9 nm were determined for the three different samples by using the BJH equation based on the desorption branch of the isotherms. The specific surface area, pore volume, and average pore size of the three kinds of hollow spheres and the commercial P25 sample are summarized in Table 1. 

From Table 1 it can be seen that all three kinds of hollow spheres show higher specific surface areas than that of the commercial P25 sample. Among the three hollow spheres, C-TiO_2_ and P-TiO_2_ show relatively higher specific surface area and pore volume than that of B-TiO_2_ sample, which indicates that both of the organic additives played important role in the introduction of pores and increase of specific surface area. C-TiO_2_ show the narrower pore size distribution than that of P-TiO_2_, which reveals that CTAB surfactant has much higher ability in pore-directing than that of PVP polymer, since the former can easily form micelles in tube-shape in the solution and has been used widely as soft template for pore introduction. N_2_ adsorption–desorption measurements further confirm the SEM and TEM results that porous TiO_2_ hollow spheres with relatively high surface area, large pore volume, and big pore size can be fabricated by using MF as core template and CTAB or PVP as pore-directing agent, particularly the former one, through an electrostatic force-assisted sol–gel deposition process.

### 3.6. XPS Analysis

**Figure 9:**
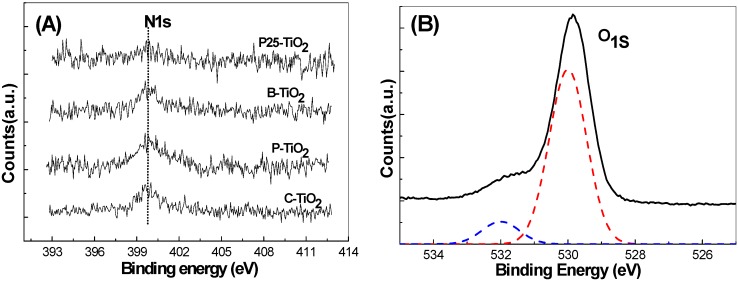
X-ray photoelectron spectroscopy (XPS) surveys of the three kinds of TiO_2_ hollow spheres in the N1s region (**A**); and the high-resolution XPS survey of C-TiO_2_ in the O_1S_ region (**B**), where the solid curve is the original survey and the two dashed-line curves show the deconvoluted peaks.

Figure 9A shows the high-resolution X-ray photoelectron spectroscopy (XPS) surveys of C-TiO_2_, P-TiO_2_, B-TiO_2_, and P25 in the N1s region. An XPS peak centered at about 400 eV can be observed for C-TiO_2_, P-TiO_2_, and B-TiO_2_ hollow spheres. It has been reported that most N species in the N-doped TiO_2_ existing in the form of nitrides, such as N in the O–Ti–N linkage, correspond to the binding energy of 399.7 eV, which is the typical N1s signal of N-doped titanium dioxide [30]. XPS results are consistent with the reported results for N-doped TiO_2_ and confirm the existence of N elements in the three hollow products [31,32]. 

XPS surveys of oxygen 1s have also been observed in all the three hollow spherical samples, Figure 9B presents the typical high-resolution XPS survey of C-TiO_2_ in the O1s region. The O1s spectra can be deconvoluted into two peaks centered at 530 and 532 eV, respectively, which can be related to the presence of oxygen and nitrogen from the same lattice units of TiO_2_ crystal. The primary peak at 530 eV can be attributed to the O–Ti–O type of oxygen coordination, whereas the small contribution at 532 eV indicates the presence of another type of oxygen due to the more covalent nature of N-doped C-TiO_2_. Similar results have also been observed for P-TiO_2_ and B-TiO_2_ samples. The atomic contents of nitrogen in B-TiO_2_, P-TiO_2_, and C-TiO_2_ mesoporous spheres, which were calculated based on the peak of the N1s, were about 0.5%, 0.9%, and 0.8%, respectively. Higher N-doping content could be achieved when CTAB or PVP were used as pore-directing agents since they can provide additional N source besides the MF template. A negligible N1s peak has been observed in the P25 XPS high-resolution survey, which might come from contaminating species. It has been reported that the doping of N atoms into the TiO_2_ lattice can introduce interbands, which then can extend the optical response of TiO_2_ materials to the low-energy light and result in the enhancement of absorption ability of this material to visible light [33].

### 3.7. Photocatalytic Properties

**Figure 10:**
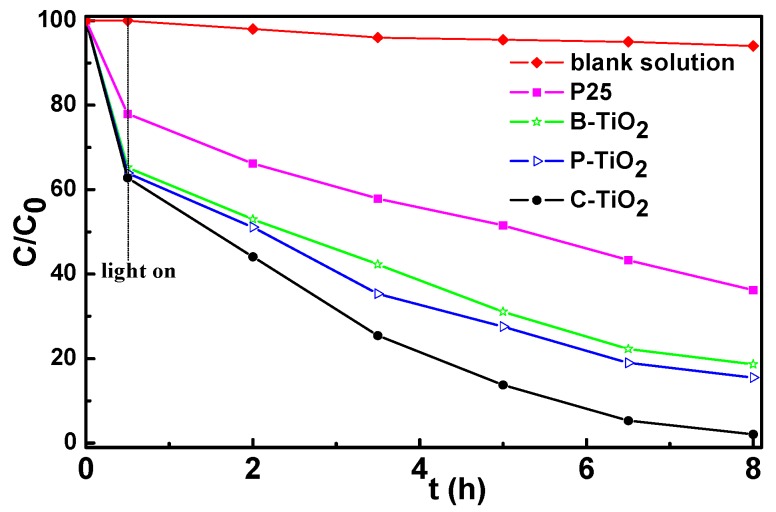
Photocatalytic activities of TiO_2_ materials under visible-light irradiation.

Figure 10 illustrates the plot of the adsorption and degradation *C*/*C*_0_ (*C*_0_ and *C* are the equilibrium concentration of MB before and after the addition of TiO_2_ hollow spheres or P25 particles, respectively) versus visible-light irradiation time. The decrease of the MB concentration before the visible-light irradiation can be attributed to the adsorption effect of the TiO_2_ particles to MB. The three hollow spheres show quite similar adsorption ability to MB, and they are much higher than that of the P25 nanoparticles due to their mesoporous properties. With light on and as irradiation time passes, the hollow spheres show much higher photocatalytic activity than that of the P25 nanoparticles. The photocatalytic activities between the hollow spheres and the P25 sample under visible-light irradiation for 7.5 h are obviously different. It could be seen that nearly 81%, 85%, 98%, and 64% of MB molecules were degraded after visible-light irradiation (wavelength >420 nm) for B-TiO_2_, P-TiO_2_, C-TiO_2_, and P25 catalysts, respectively. B-TiO_2_ and P-TiO_2_ hollow spheres show similar photocatalytic behavior. Interestingly, C-TiO_2_ exhibited the photocatalytic activity superior to the other two mesoporous TiO_2_ hollow spheres (B-TiO_2_, P-TiO_2_) and the commercial P25 for the degradation of MB in the visible-light region. Distinctly, the MB concentration changed slightly under visible-light irradiation without a TiO_2_ catalyst, indicating that the presence of a TiO_2_ catalyst is necessary for photocatalytically decomposing MB. From the analysis we can see that the photocatalytic activities of theses samples are related to their specific surface area. Then, one may speculate that the reason for the faster degradation of MB on the hollow microspheres is due to their high specific surface area, which could simply also provide a larger number of reaction sites than the solid P25. However, the influencing factors on the photocatalytic properties of the TiO_2_ matrices are complicated; other possibilities such as exposed lattice facet, light harvest ability, and so on can also affect their photocatalytic activity [34]. An endeavor to illustrate this issue is still underway in our group. 

### 3.8. Optical Properties

**Figure 11:**
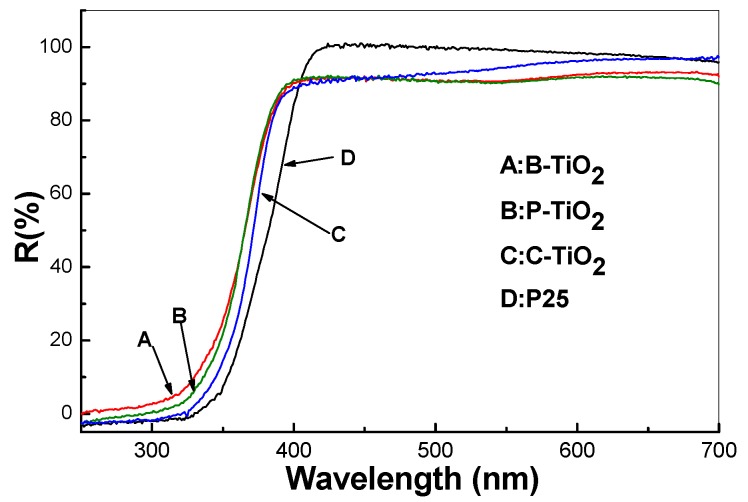
Diffusion reflectance spectra of the mesoporous TiO_2_ hollow spheres (**A**: B-TiO_2_, **B**: P-TiO_2_, **C**: C-TiO_2_, and the commercial P25 titania **D**.

Figure 11 depicts the diffusion reflectance spectra of the mesoporous hollow spheres of B-TiO_2_, P-TiO_2_, C-TiO_2_, and the commercial P25 particles, respectively. From the figure it can be seen that there are two kinds of distinctions among them. The first one is the absorption thresholds of the four curves, from which one can calculate the bandgaps of the samples. The second one is their transmittance properties between 400 and 700 nm, which is exactly in the visible light range. In comparison of P25 with B-TiO_2_, P-TiO_2_, and C-TiO_2_ hollow spheres, it can be seen that the absorption thresholds of samples B-TiO_2_, P-TiO_2_, and C-TiO_2_ show obvious blue-shift, which is contrary to our expectations that the N-doping will introduce interbands to the TiO_2_ crystals and induce red-shift of their light absorption thresholds.

Nevertheless, all curves of the three hollow spheres show steeper slopes than that of the P25 TiO_2_, indicating the size of the particles composing the hollow spheres is nearly monodispersive. From the absorption thresholds, one can perceive intuitively the remarkable difference among the bandgaps of the four samples. Moreover, a quantitive estimate of the optical bandgaps can be obtained using the following equations based on the reflection spectra [35]:

(1) α(ν) = *A*(h′ν − *Eg*)^*m*/2^

where h′ = h/2π, h is the Planck constant, h′ν is the photon energy, α is the absorption coefficient, while m is dependent on the nature of the transition. For a direct transition m is equal to 1 or 3, whereas for an indirect allowed transition *m* is equal to 4 or 6. Since α is proportional to *F*(*R*), the Kubelka–Munk function *F*(*R*) = (1 − *R*)^2^/2*R*, the energy intercept of a plot of (*F*(*R*) × hν)^1/2^ versus h′ν gives *Eg* for an indirect allowed transition when the linear region is extrapolated to the zero ordinate [36].

**Figure 12:**
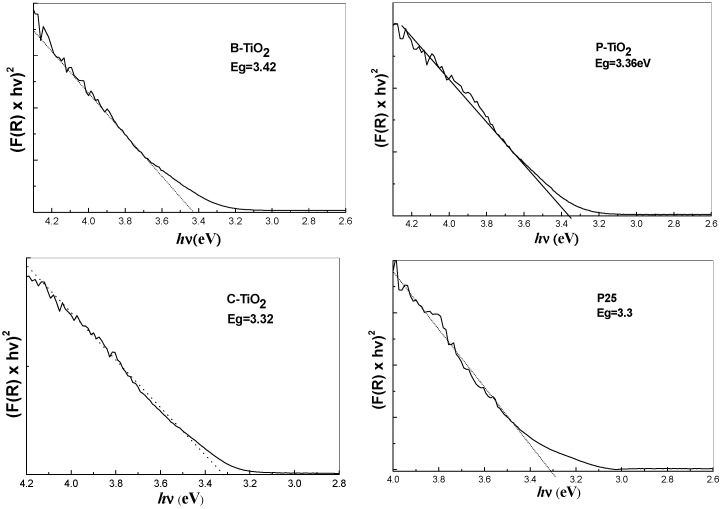
The normalized plots derived from the calculated data using Equation (1) based on the absorption data of Figure 11.

The normalized plots derived from the calculated data based on the four curves in Figure 11 are depicted in Figure 12, respectively. Similar results are obtained if (*F*(*R*) × hν)^2^ is plotted against h′ν, as is appropriate for a direct semiconductor. For the present comparative exercise, however, we have opted to use the equation for an indirect semiconductor in accordance with common practice. Bandgaps of 3.42, 3.36, and 3.32 eV were obtained for samples B-TiO_2_, P-TiO_2_, and C-TiO_2_, respectively, which are larger than that of the bulk crystalline TiO_2_ (the room temperature bulk bandgaps for anatase TiO_2_ and rutile TiO_2_ are 3.2 and 3.0 eV, respectively) [37]. 

The optical property of commercial P25 has also been detected for comparison, and a bandgap of about 3.3 eV is obtained. The calculated bandgaps fit previous values obtained for TiO_2_ particles with sizes between several and 10 nanometers [38,39]. Therefore, the blue-shift of the absorption thresholds for samples B-TiO_2_, P-TiO_2_, and C-TiO_2_ consisting of the same phase of TiO_2_ nanocrystals can be attributed to the quantization effect of the nanoscale particles [40], which may counteract the influence of nitrogen-doping on the bandgap of the titania spheres, even though the XPS measurements demonstrated that samples B-TiO_2_, P-TiO_2_, and C-TiO_2_ are doped with nitrogen atoms [41]. From the comparison we could deduce that the porous character and the hollow property of the integrated TiO_2_ matrix can not affect the inherent bandgap of the TiO_2_ crystals, while they might enhance the light harvest through multiscattering and reabsorption of the initial incident light. The absorption ability in the visible region of the UV–vis spectra can be attributed to the scattering effect of the cavities inside the matrices. Meanwhile, the porous character will influence further the scattering ability of the porous TiO_2_ shell by modifying their refractive indices [42].

## 4. Conclusions

N-doped hollow TiO_2_ spheres with a porous wall have been successfully fabricated through a sol–gel synthesis using MF microspheres as sacrificial template. Porous TiO_2_ hollow spheres with size from 0.4 to 1.2 μm and wall thickness between 40 and 110 nm are derived from the corresponding core–shell microspheres by calcinations. The MF spheres play two roles in the preparation: (1) as the template for introducing the hollow caves and (2) the nitrogen source for doping the N atoms into the TiO_2_ nanocrystals. The addition of CTAB or PVP additives can induce large pore volume and more regular pores, and they can also enhance the N-doping content due to the N atom included in their molecules. The porous TiO_2_ hollow spheres show higher photocatalytic activities than that of the commercial P25 titania. Among the three kinds of porous TiO_2_ hollow spheres, the C-TiO_2_ sample show much superior photocatalytic activity than the other two hollow spheres. The doping of the N atoms in the lattice of the TiO_2_ nanocrystals composed of the hollow spheres has been verified by XPS measurements. No concrete evidence points to the N-doping or the doping content as the cause of the enhancements of the photocatalytic activities of the porous TiO_2_ hollow spheres. In a word, the photocatalytic efficiency of the TiO_2_-based matrix can be higher than that of the pristine TiO_2_ particles by reasonably modifying the composition of the materials and by rationally designing the structure of the integrated TiO_2_ nanocrystals matrix; for instance, the construction of porous hollow spherical structure.

## Figures and Tables

**Table 1:** Specific surface area, pore volume, and pore-size distribution of the samples.

Sample Name	Specific Surface Area	Pore Volume	Average Pore Width
P25	50	- ^a^	- ^b^
B-TiO_2_	98	0.13	8.7
P-TiO_2_	128	0.30	10.6
C-TiO_2_	131	0.41	12.9

^a,b^ the values are unavailable due to the compact property of P25 TiO_2_ particles.
